# *Plasmodium falciparum* Gametocyte Enrichment in Peripheral Blood Samples by Magnetic Fractionation: Gametocyte Yields and Possibilities to Reuse Columns

**DOI:** 10.4269/ajtmh.18-0773

**Published:** 2019-01-02

**Authors:** Wouter Graumans, Chiara Andolina, Shehu S. Awandu, Lynn Grignard, Kjerstin Lanke, Teun Bousema

**Affiliations:** 1 Department of Medical Microbiology, Radboud University Nijmegen Medical Centre, Nijmegen, The Netherlands;; 2 Department of Immunology and Infection, London School of Hygiene and Tropical Medicine, London, United Kingdom

## Abstract

Gametocytes are sexual stage malaria parasites responsible for transmission to mosquitoes. Multiple gametocyte-producing clones may be present in natural infections, but the molecular characterization of gametocytes is challenging. Because of their magnetic properties, gametocyte enrichment can be achieved by magnetic fractionation. This increases detection sensitivity and allows specific genotyping of clones that contribute to malaria transmission. Here, we determined the percentage of *Plasmodium falciparum* gametocytes successfully bound to magnetic activated cell sorting (MACS) LS columns during magnetic fractionation and assessed whether columns can be reused without risking contamination or affecting column binding efficiency. Bound column fractions were quantified using multiplex quantitative reverse transcription polymerase chain reaction (qRT-PCR) for male (pfMGET) and female (CCp4) gametocytes and ring-stage asexual parasites (SBP1). To investigate cross contamination between columns, parasite strain identity was determined by merozoite surface protein 2 genotyping followed by capillary electrophoresis fragment sizing. A reproducible high percentage of gametocytes was bound to MACS LS columns with < 5% gametocytes appearing in the flow-through and < 0.6% asexual ring-stage parasites appearing in the gametocyte fraction. A high yield (> 94%) of gametocyte enrichment was achieved when columns were used up to five times with lower binding success after eight times (79%). We observed no evidence for cross contamination between columns.

## INTRODUCTION

Ongoing malaria transmission poses a considerable public health burden in endemic countries worldwide.^1^ Caused by protozoan parasites of the *Plasmodium* genus, six species are able to infect human hosts on the bite of infected female *Anopheles* mosquitoes. Mosquitoes are born uninfected and may become infected after ingestion of blood meals containing circulating mature sexual stages of the parasite, known as gametocytes. Once ingested by the mosquito, gametocytes fertilize and, following sporogonic development, produce hundreds to thousands sporozoites that migrate to the salivary glands of the mosquito and render the mosquito infectious to humans on their next bite.

Malaria transmission potential is influenced by many factors including gametocyte density^2^ and several parasite factors that determine gametocyte fitness. Several studies exploited the paramagnetic properties of gametocytes and used magnetic column fractionation to assess gametocyte prevalence and density in peripheral blood samples before and after antimalarial treatment.^3–6^ Parasites use intraerythrocytic hemoglobin as main protein source for development. Heme, a by-product after digestion, is oxidized and converted in hemozoin, also known as malaria pigment.^7,8^ Hemozoin accumulates over time in infected erythrocytes and has paramagnetic properties in late-stage trophozoites, schizonts, and gametocytes.

Magnetic column fractionation can be used to increase parasite detectability.^9^ This may be particularly useful for gametocytes that commonly circulate at low densities,^10^ sometimes too low to infect mosquitoes.^11^ Magnetic fractionation allows specific genotyping of asexual and gametocyte fractions to identify the following: signatures of drug resistance,^12^ the number of acquired *Plasmodium* clones over time (known as the force of infection),^13^ the multiplicity of infection (MOI; the number of parasite clones present in a blood sample), and which clones contribute to malaria transmission.^14^ Although molecular detection of gametocytes without magnetic concentration is possible and operationally attractive in well-equipped laboratories, it has limitations in terms of the genetic characterization of gametocytes because gametocytes only comprise a small fraction of the total parasite biomass and there are few suitable polymorphic gametocyte RNA targets.^15^ Assessing gametocyte MOI and resistance markers is of interest because MOI may affect transmission success^16,17^ and drug-resistant parasite clones may have a transmission advantage compared with wild-type parasites.^18^

In the present study, MACS cell separation columns based on magnetic binding were used to capture mature gametocytes and separate these from asexual parasites. Concentrated gametocytes were used to quantify their density and assess their molecular signature. To make the assay more attractive for large-scale studies, a procedure to wash and reuse columns was validated in terms of gametocyte yield and risks of contamination. For quantification, a qRT-PCR was used to quantify messenger RNA from asexual parasite rings (SBP1) and male (pfMGET) and female (CCp4) gametocyte-specific genes.^19,20^ Parasite strain identity was determined by merozoite surface protein 2 (MSP2) genotyping followed by capillary electrophoresis (CE) fragment sizing.^21,22^

## MATERIALS AND METHODS

### Culture, synchronizing, and dilution of *Plasmodium falciparum* parasites.

An automated culture system was used^23^ to culture the Nijmegen *falciparum* (NF) 54 (West-Africa), NF135 (Cambodia), and a previously not presented NF175 (Nigeria) strain.^24,25^ The medium was replaced automatically twice a day and asexual replication was maintained by suspension of 0.5% parasites in 5% red blood cells every 2–3 days. Cultures were ring synchronized with 5% sorbitol in H_2_O. For gametocyte production, asynchronous cultures were started on day 0 (0.5% parasites, 5% red blood cells) and treated with N-acetyl-glucosamine (50 mM; Sigma Aldrich, Darmstadt, Germany) from day 9 to 14 to stop off asexual parasite replication. Cultures were harvested at day 16 (0.3–0.5% gametocytes and 2% hematocrit).

For asexual parasite cultures, Giemsa’s stained blood smears were made to determine the percentage of infectivity (Supplemental Figure 1). A 100 times dilution of culture material in phosphate buffered saline (PBS) (1×) was made to count the number of erythrocytes with a hemocytometer. The equivalent volume for 70,000 ring-stage parasites per µL was added to 0.5 mL of peripheral fresh collected ethylenediaminetriacetric acid (EDTA) blood (Becton Dickinson, Vacutainer system).

**Figure 1.**
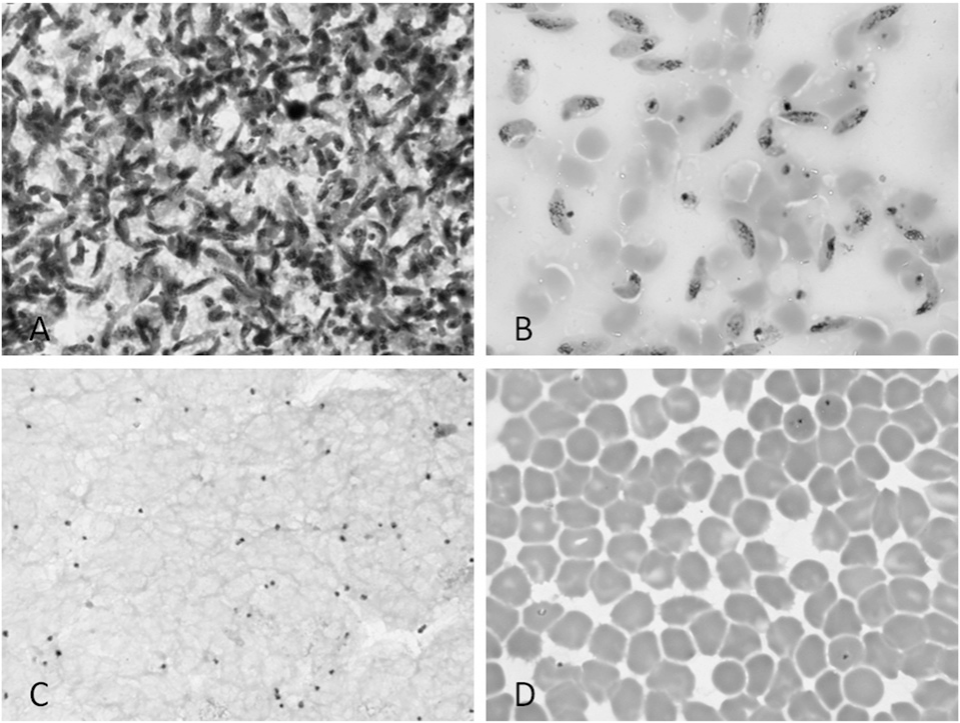
Stained blood smears of bound and flow-through fraction. Images are Giemsa’s stained blood smears using light microscopy at 1,000 magnification. Thick smear and blood smear of bound fraction (**A** + **B**) and flow-through fraction (**C** + **D**).

For N-acetyl-glucosamine–treated gametocyte cultures, blood smears were Giemsa stained at day 16 to confirm the absence of asexual parasites and presence of stage five gametocytes (Figure 1). No evidence of gametocyte activation (exflagellating or the production of female round forms) was observed in any of the slides examined. The total number of gametocytes per milliliter culture was counted with a hemocytometer. The equivalent volume of the desired end concentration was transferred to a 15 mL tube. Tubes were spun down (2,000 rpm, 5 minutes) and supernatant was removed; a volume of 2.5 mL was left behind. Subsequently, 0.5 mL preheated uninfected EDTA blood was added.

### MACS procedure and washing of columns.

The QuadroMACS separator (Miltenyi Biotec, Bergisch Gladbach, Germany) with LS columns was used, inside a 37°C incubator, to capture gametocyte-infected red blood cells. All to be used materials were preheated to 37°C. A 25G hypodermic needle (Becton Dickinson, Franklin Lakes, NJ) was attached to each column to reduce flow speed to 0.67 mL per minute (Supplemental Figure 2A and B). Up to four columns were attached to the MultiStand and 15 mL tubes were placed underneath to collect flow-through material. To hydrate the columns, 1 mL of Roswell Park Memorial Institute (RPMI) medium was added (Supplemental Figure 2C and D) and after the flow ceased, a premix of 2.5 mL RPMI with 0.5 mL of infected or uninfected peripheral EDTA blood was added (Supplemental Figure 2E). After all material flowed through (Supplemental Figure 2F and G), columns were washed two times with 1 mL of RPMI medium to remove unbound cells (Supplemental Figure 2H and I). Needles were removed and columns were detached from the MultiStand and transferred to new 15 mL tubes. Two times 1 mL of RPMI medium was added to the columns to elute bound material. After the flow ceased, the plunger was used to remove the last remaining medium. All tubes, bound fraction and flow-through fraction, were spun down at 2,000 rpm in a prewarmed 37°C centrifuge. The supernatant was removed and five times the equal volume of RNA protect was added (Qiagen, Hilden, Germany). The sample was frozen at −80°C to be extracted later.

For reusage experiments, columns were washed five consecutive times with distilled water and the plunger was used each time to flush out the water. This procedure was repeated five times with 70% ethanol. Washed columns were dried overnight in an incubator at 37°C and reused the following day.

### Parasites quantification and identification by MSP2 genotyping and qRT-PCR.

Total nucleic acid (NA) was extracted with the automated MagNA Pure LC instrument (Roche, Basel, Switzerland) using the MagNA Pure LC Total Nucleic Acid Isolation Kit—High Performance (Roche) and eluted in 50 μL. Samples were used immediately or stored at −20°C for long term storage. To distinguish parasites molecularly, the single copy polymorphic MSP2 gene was used for genotyping. In a nested PCR, first the entire MSP2 gene segment was amplified and subsequently used to amplify the allele strain-specific gene variant type FC27 or 3D7.^26^ Fluorescently labeled primers were used to enable laser detection after capillary gel electrophoresis; Peak Scanner version 2 software (Applied Biosystem, Foster City, CA) was used for automated fragment sizing.^21,22^

For parasite quantification, isolated messenger RNA (mRNA) was used in a qRT-PCR multiplex assay for pfMGET (males) and CCp4 (females) transcripts for the gametocytes^27,28^; a separate qRT-PCR was used for asexual parasites (SBP1).^20^

### MACS column binding efficiency of cultured gametocytes, with and without asexual ring stages.

To determine the binding efficiency of MACS columns, a 10-fold serial dilution of NF54 gametocyte culture material was made in 0.5 mL of peripheral EDTA blood in triplicate (gametocyte starting conc. 4,000/µL). The column bound fraction and flow-through fraction were collected for qRT-PCR analysis. In an identical experimental setup, 70,000/µL NF54 asexual ring-stage parasites were added to each of the serial dilution samples to mimic a natural infection (gametocyte start conc. 10,000/µL). The column bound fraction and flow-through fraction were collected for qRT-PCR analysis.

### Validating reusage of MACS columns by MSP2 genotyping and qRT-PCR.

In a three-way approach, we tested whether columns can be reused without risking contamination or affecting column binding efficiency.

In the first set, new columns were used to capture 10,000 gametocytes/µL cultured NF54 gametocytes in a total volume of 0.5 mL of peripheral EDTA blood per column. The column bound fractions and flow-through fractions were collected for MSP2 analysis. Columns were reused the following day after washing procedure and 0.5 mL uninfected EDTA blood was run as a negative control. Samples were collected for MSP2 analysis. The procedure was repeated for a second (NF135) and third strain (NF175), all performed in triplicate.

In the second set, new columns were used to capture 10,000 gametocytes/µL in 0.5 mL of peripheral EDTA blood. In triplicate, the NF54, NF135, and NF175 strains were processed on the same column, with washing in between. The bound fractions and flow-through fractions were collected for MSP2 analysis.

For the third set, a new column was used to capture cultured NF54 gametocytes in a total volume of 0.5 mL of peripheral EDTA blood. Bound fractions and flow-through fractions were collected for qRT-PCR analysis. Columns were washed, and the next day, the same procedure was repeated with 0.5 mL of uninfected EDTA blood as negative control. Samples were collected for qRT-PCR analysis and the whole procedure was performed another four times.

### Analysis.

Merozoite surface protein 2 analysis of samples was initially performed by running an agarose gel to visualize positive and negative samples from nested PCR products. All the products were subsequently processed by CE and different MSP2 alleles analyzed by Peak Scanner software. Size, dye color, and height were used to define the different alleles in the 3D7 and FC27 families.

For qRT-PCR, the number of parasites per milliliter was calculated with a standard curve, obtained using 10-fold dilutions of cultured gametocytes (for CCP4 and pfMGET) or asexual parasites (for SBP1) (Supplemental Figure 3).

## RESULTS

### Cultured gametocytes are retained with high yield by MACS columns.

To determine the efficiency of parasite binding on MACS columns, two 10-fold serial dilutions with five concentrations were made in triplicate with starting concentrations of 4,000 gametocytes/µL (I) and 10,000 gametocytes/µL (II) and quantified by qRT-PCR. Both showed excellent agreement between estimated parasite density by serial dilution and qRT-PCR (mean *R*^2^ 0.995, range 0.984–0.999).

**Figure 2.**
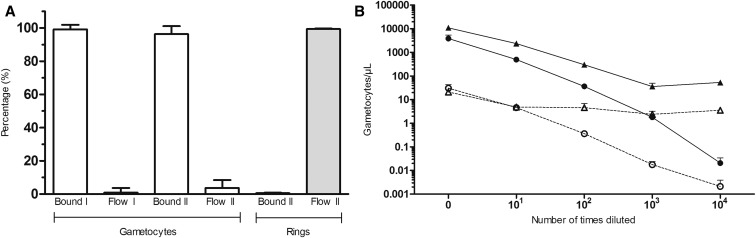
MACS column gametocyte and asexual ring stages binding efficiency quantified by qRT-PCR. The mean percentage of gametocytes and asexual parasites appearing in the bound fraction or flow-through fraction is shown for two independent serial dilutions that were both examined in triplicate. Starting concentrations for series I and II were 4,000 and 10,000 gametocytes/µL, respectively. Panel **A** shows parasite populations as a fraction of the input material and panel **B** shows the total number of gametocytes per µL for the bound fraction (solid line) or flow-through fraction (dotted line) of series I (circles) and II (triangles).

To demonstrate binding efficiency, the quantified gametocytes in the bound and flow-through fractions were shown in percentages (Figure 2A) and absolute numbers (Figure 2B). For dilution I, the mean proportion of gametocytes that were bound was 99.11% (*n* = 15, range 89.02–100, SD 2.8) and the mean proportion of gametocytes that were detected in the unbound flow-through fraction was 0.886% (*n* = 15, range 0–10.98, SD 2.8). For dilution II, the mean proportions were 96.33% (*n* = 14, range 82.71–99.86, SD 4.77) and 3.673% (*n* = 14, range 0.14–17.29, SD 4.77), respectively. In dilution II, asexual ring-stage parasites were added to the infected blood at a concentration of 70,000 par/µL to mimic a natural infection density (Figure 2A). The proportions of ring-stage parasites in the bound column fraction and flow-through fraction were 0.64% (*n* = 14, range 0.23–0.946, SD 0.215) and 99.36% (*n* = 14, range 99.05–99.77, SD 0.215), respectively.

### Cross contamination and binding efficiency when MACS columns are reused.

**Figure 3.**
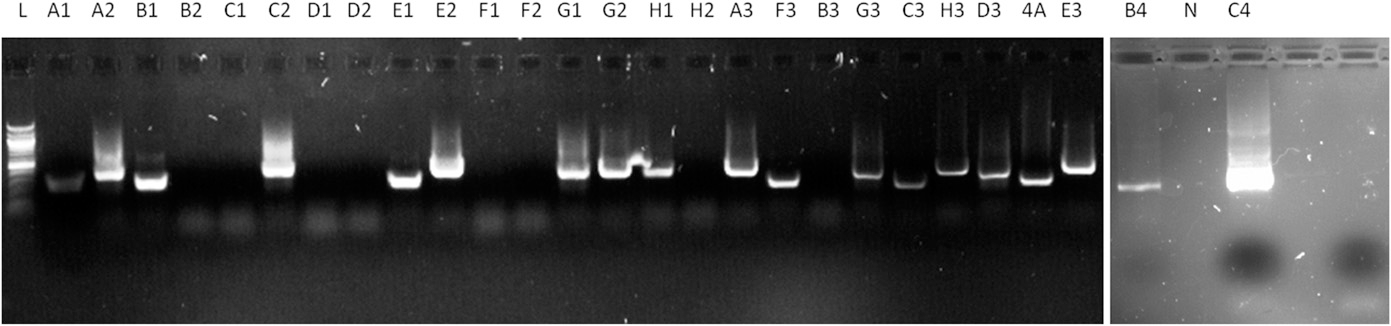
Merozoite surface protein (MSP2) MACS-bound column fraction results for infected and uninfected blood with overnight column washing in between. Gel electrophoresis image results of MSP2 genotyping from collected bound column fractions. Nijmegen *falciparum* (NF)-54, NF135, and NF175 can be can be distinguished on difference in fragment size. All negative samples run with uninfected blood were negative, and show no result on gel.

To investigate cross contamination in reused columns, we first performed experiments where a column was used to bind gametocytes, washed, and subsequently used with infected or uninfected blood. Three genetically distinct parasite lines showed distinguishable MSP2 PCR products after gel electrophoresis (Figure 3) and CE (Supplemental Tables 1 and 2), confirming the expected parasite genotype in bound fractions. Of the experiments with infected–uninfected blood, one sample showed PCR-detectable parasite material (Supplemental Table 1). A total of 17 of 18 analyzed samples thus had matching results. In the experiments that repeatedly used infected blood, all gel electrophoresis results matched the expected result (9/9), but CE failed for one sample (8/9 matching results) (Supplemental Table 2).

**Figure 4.**
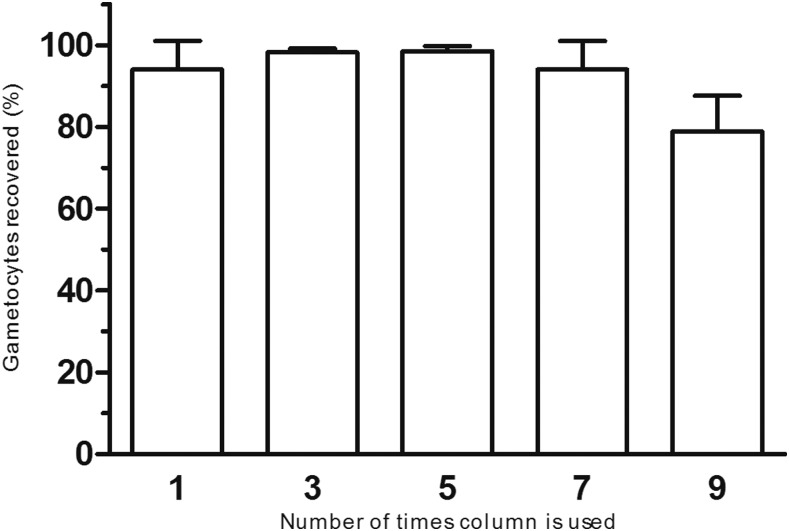
MACS column validation by qRT-PCR for reusage by column washing up to five times. In the column bar graph, an experimental triplicate is shown of three new columns used multiple times, with column washing in between. For runs 1, 3, 5, 7, and 9, gametocyte in vivo culture material was used to determine column binding efficiency over time. For runs 2, 4, 6, 8, and 10, negative blood was used to investigate cross contamination after washing (all negative, not shown). The number of gametocytes quantified by qRT-PCR of the bound column fraction is shown in percentage (%). The measured number of gametocytes in runs 1, 3, 5, 7, and 9 was 4,525 (range 2,572–6,294), 2,482 (1,108–6,338), 3,828 (1,250–7,453), 7,561 (4,051–12,230), and 2,210 (1,171–3,650), respectively.

To investigate the binding efficiency and potential contamination following reusing of columns, gametocyte qRT-PCR was performed to determine the number of gametocytes present in the bound fraction and flow-through fraction. Columns were used up to 10 times, alternating positive gametocyte samples on 1 day and negative blood samples on the next day. Columns were washed in between, and dried overnight to be used the following day. The binding efficiencies of the columns for the positive samples over time were as follows: Run 1. mean 94.01% (*n* = 3, range 86.17–99.59, SD 6.99), Run 3. mean 98.24% (*n* = 2, range 97.63–98.85, SD 0.86), Run 5. mean 98.46% (*n* = 3, range 96.96–99.41, SD 1.317), Run 7. mean 94.04% (*n* = 3, range 86.06–98.48, SD 6.93), and Run 9. mean 78.90% (*n* = 3, range 71.04–88.17, SD 8.65). Runs 2, 4, 5, 8, and 10 were performed with negative blood and were negative by qRT-PCR (Figure 4). The number of bound gametocytes is given in the legend of Figure 4.

## DISCUSSION

The aims of this study were to determine the proportion of *P. falciparum* gametocytes successfully bound to MACS LS columns during magnetic fractionation and to assess whether columns can be reused without risking contamination or affecting column binding efficiency.

Our findings demonstrate that a high percentage of gametocytes is bound to MACS LS columns with < 5% gametocyte appearing in the flow-through. Karl et al.^29^ previously reported no variation between *P. falciparum* parasite strains in MACS binding capacity and efficient binding at densities less than 1 gametocyte/µL.^30^ In the present study, we complement these findings and mimic natural infections, where gametocytes typically comprise < 10% of the total parasite biomass,^10^ by adding an excess of asexual ring-stage parasites to our gametocyte material. Ring-stage asexual parasites are the major other parasite stage in the circulation, schizonts being observed very rarely in the peripheral blood of malaria patients and mostly in severe cases with very high parasite burden.^31,32^ As expected, only a small proportion of rings (0.6%) was found in the bound fraction without a disadvantageous influence of asexual parasites on the gametocyte binding efficiency of columns. A small proportion of young-stage schizonts that survived sorbitol treatment was observed in Giemsa’s stained smears with a ring/schizont ratio of 150:1 (0.67%) and may have been responsible for the low-level binding of asexual parasites to the column. From this, we conclude that concentrating gametocytes up to 10,000 parasites/µL per 0.5 mL of peripheral blood is highly efficient and very reproducible. This supplements previous reports that the highest capture efficiencies are achieved at a minimal flow rate and minimal concentration of infected cells and large blood volumes are not affecting gametocyte binding efficiency.^29^ Our detection of male and female gametocytes suggests that also gametocyte sex ratio can be sensitively assessed following MACS enrichment; we found no evidence for a loss of exflagellating male gametocytes during the MACS procedures that could have distorted sex ratio assessments.

Reusage of columns is operationally and financially attractive, especially for field laboratories. In the present study, high gametocyte quantities were processed on MACS columns with intermediate washing steps. Collected bound gametocyte fractions were analyzed by MSP2 PCR and gel/CE to identify potential cross contamination. For gel electrophoresis, 26 of 27 samples matched expected results, and for CE, 25 of 27 samples. Failures were plausibly due to a incidental error in the washing procedure or contamination during DNA isolation and a technical failure in CE. In the multiplex qRT-PCR, we observed a consistent high yield of gametocytes (> 94%) when columns were used up to five times but lower binding success after eight times (79%). There was a reduced flow speed after usage of the column five times and a few dots of rust appearing after usage of seven times, but this had no apparent influence on the binding efficiency.

Our findings build on earlier observations that magnetic fractionation of blood from natural infections can increase the sensitivity of gametocyte detection^30,33^ and that it is highly challenging to characterize gametocyte producing clones based on polymorphic mRNA targets.^15^ In the present study, we demonstrated that magnetic fractionation may be used to overcome this problem by concentrating gametocytes from (large) blood volumes and subsequent conventional genotyping approaches. This enables the detection of gametocyte clonal complexity^14^ and potentially a more detailed examination of gametocyte production in relation to antimalarial drug resistance.^34^ The latter may be particularly relevant because antimalarial drug resistance may differentially impact asexual parasite survival, gametocyte production and transmission,^35–37^ and sensitively examining the molecular signatures in asexual, and gametocyte fractions may thus help in understanding how drug-resistant parasites spread and respond to drug pressure.

## Supplementary Files

Supplemental tables and figures
