# Supplementary material for: Plasmodium falciparum Gametocyte Enrichment in Peripheral Blood Samples by Magnetic Fractionation: Gametocyte Yields and Possibilities to Reuse Columns
Source: Am J Trop Med Hyg. 2019 Jan 2;100(3):572–7. doi: 10.4269/ajtmh.18-0773 (PMC6402936; doi:10.4269/ajtmh.18-0773)

The following are supplemental materials and will be published online only

Supplemental Table S1: Parasite genotyping results by MSP2 analysis of bound MACS column fractions. For three strains in triplicate gametocyte infected blood was run over a new column and after column washing the next day uninfected blood was run. The experiment was performed in triplicate.

		**Column concentration (par./µl)**	**MSP2 Gel electrophoresis (pos + /neg -)**		**MSP2 capillary gel electrophoresis (fragment size in basepair)**
**NF54 (A)**	New column	10,000	A1	+	Positive 259 bp (3D7)
	Washed column	0	C1	-	Negative
**NF54 (B)**	New column	10,000	B1	+	Positive 259 bp (3D7)
	Washed column	0	D1	-	Negative
**NF54 (C)**	New column	10,000	E1	+	Positive 259 bp (3D7)
	Washed column	0	F1	-	Negative
**NF135 (A)**	New column	10,000	G1	+	Positive 338 bp (3D7)
	Washed column	0	H1	+	Positive 338 bp (3D7)
**NF135 (B)**	New column	10,000	A2	+	Positive 338 bp (3D7)
	Washed column	0	B2	-	Negative
**NF135 (C)**	New column	10,000	C2	+	Positive 338 bp (3D7)
	Washed column	0	D2	-	Negative
**NF175 (A)**	New column	10,000	E2	+	Positive 411 bp (FC27)
	Washed column	0	F2	-	Negative
**NF175 (B)**	New column	10,000	G2	+	Positive 411 bp (FC27)
	Washed column	0	H2	-	Negative
**NF175 (C)**	New column	10,000	A3	+	Positive 411 bp (FC27)
	Washed column	0	B3	-	Negative

Supplemental Table S2: MSP2 MACS bound column fraction results with three different parasite strains on the same column and overnight column washing in between

			Concentration (par./µl)	MSP2 Gel electrophoresis (pos + /neg -)		MSP2 capillary gel electrophoresis (fragment size in basepair)
**NF54**		New column	10,000	C3	+	Positive 259 bp (3D7)
**NF135**	**A**	Washed column	10,000	D3	+	Positive 338 bp (3D7)
**NF175**		Washed column	10,000	E3	+	Negative
**NF54**		New column	10,000	F3	+	Positive 259 bp (3D7)
**NF135**	**B**	Washed column	10,000	G3	+	Positive 338 bp (3D7)
**NF175**		Washed column	10,000	H3	+	Positive 411 bp (FC27)
**NF54**		New column	10,000	A4	+	Positive 259 bp (3D7)
**NF135**	**C**	Washed column	10,000	B4	+	Positive 338 bp (3D7)
**NF175**		Washed column	10,000	C4	+	Positive 411 bp (FC27)

Supplemental Figure S1: *P. falciparum* gametocyte stained blood smears of in vivo cultures

Giemsa's stained blood smears of *P. falciparum* in vivo cultures, light microscopy 1,000x magnification. Unsynchronized culture at day 16 with multiple parasites stages (A). NF54 gametocyte N-acetyl-glucosamine treated culture, day 14 (B) and day 16 (C). Ring stage culture after sorbitol treatment (D). Day 16 gametocyte N-acetyl-glucosamine treated cultures of NF135 (E) and NF175 (F).

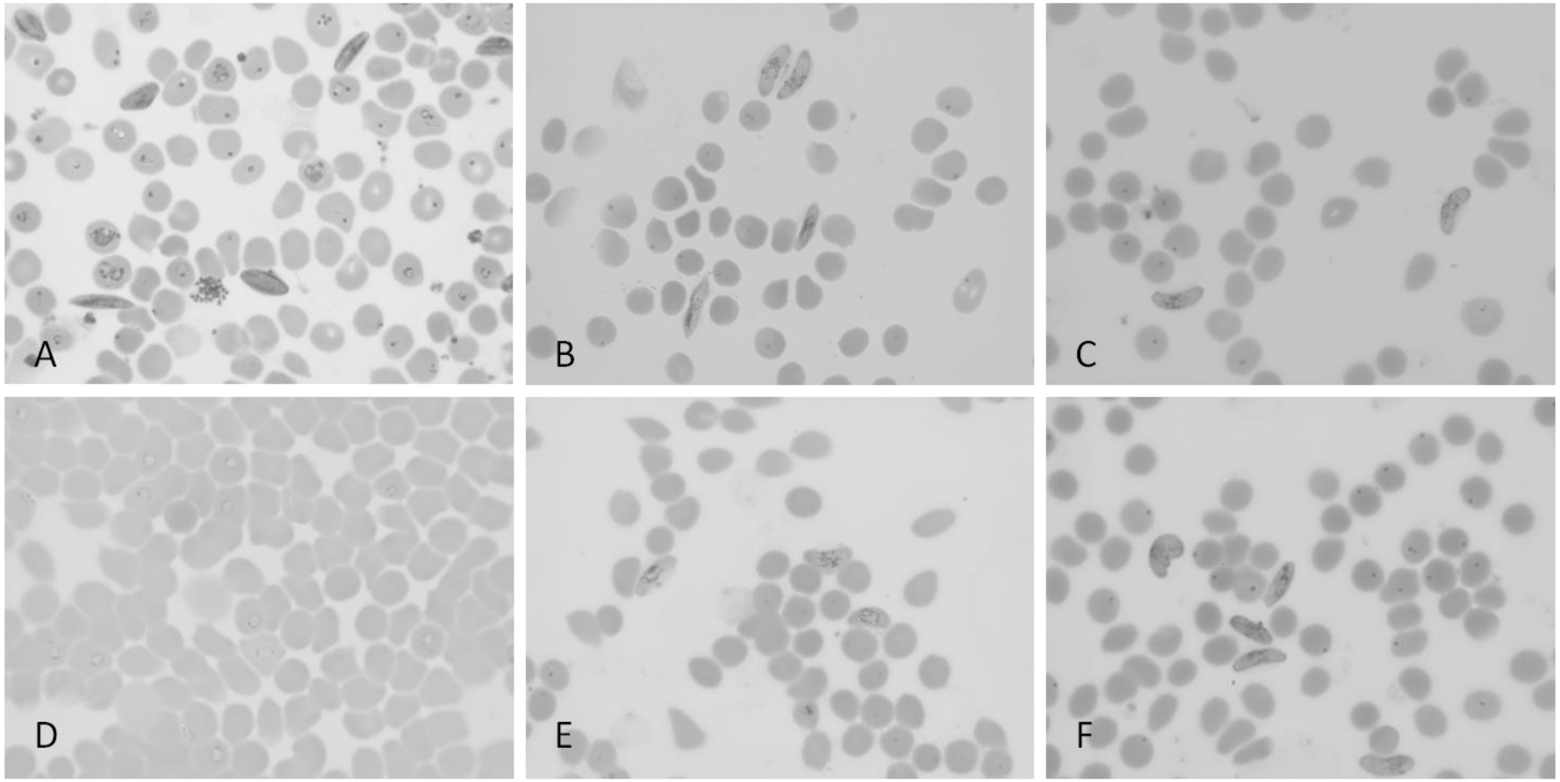

Supplemental Figure S2. MACS Column Procedure

A QuadroMACS separator (Miltenyi Biotec) with up to four LS columns was used, with a hypodermic needle attached to reduce the flow speed (A+B). Columns were hydrated with RPMI medium (C+D), accordingly peripheral infected or uninfected EDTA blood was added (E). After all material flowed through (F+G), columns were washed two times with RPMI medium to remove unbound red blood cells (H+I).

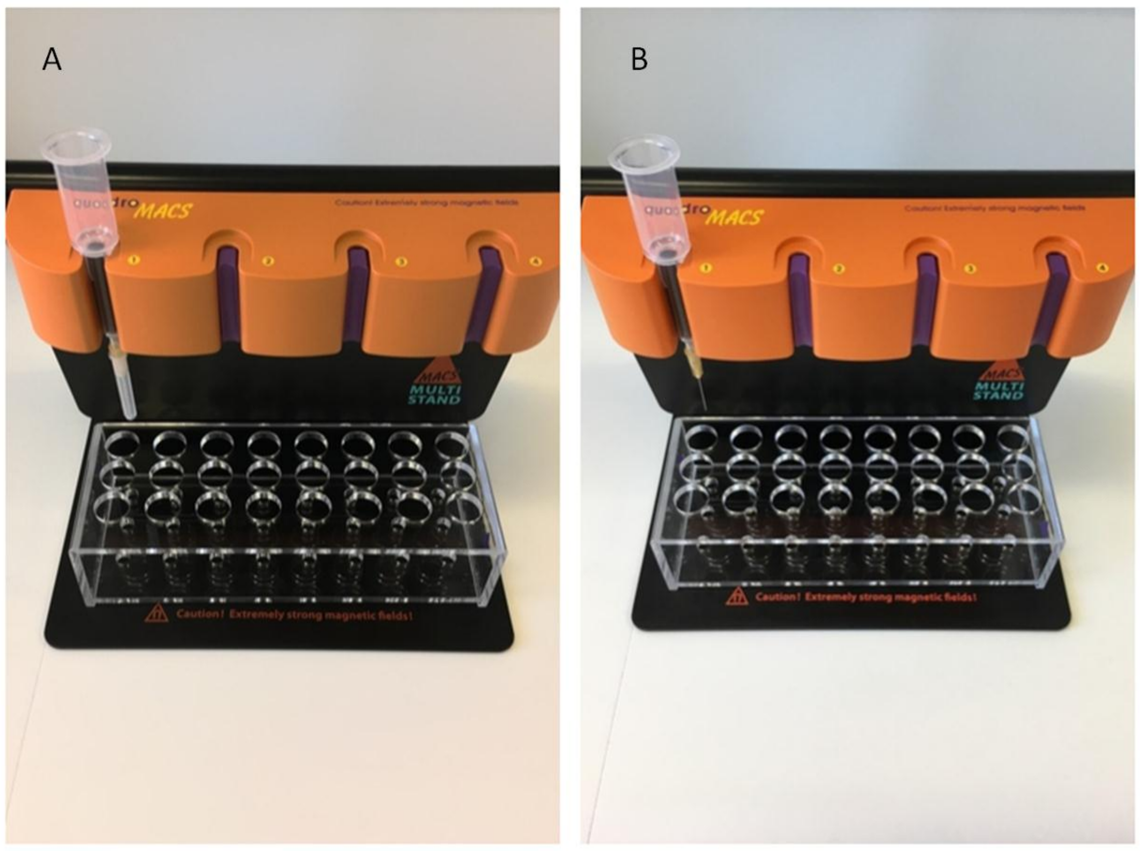

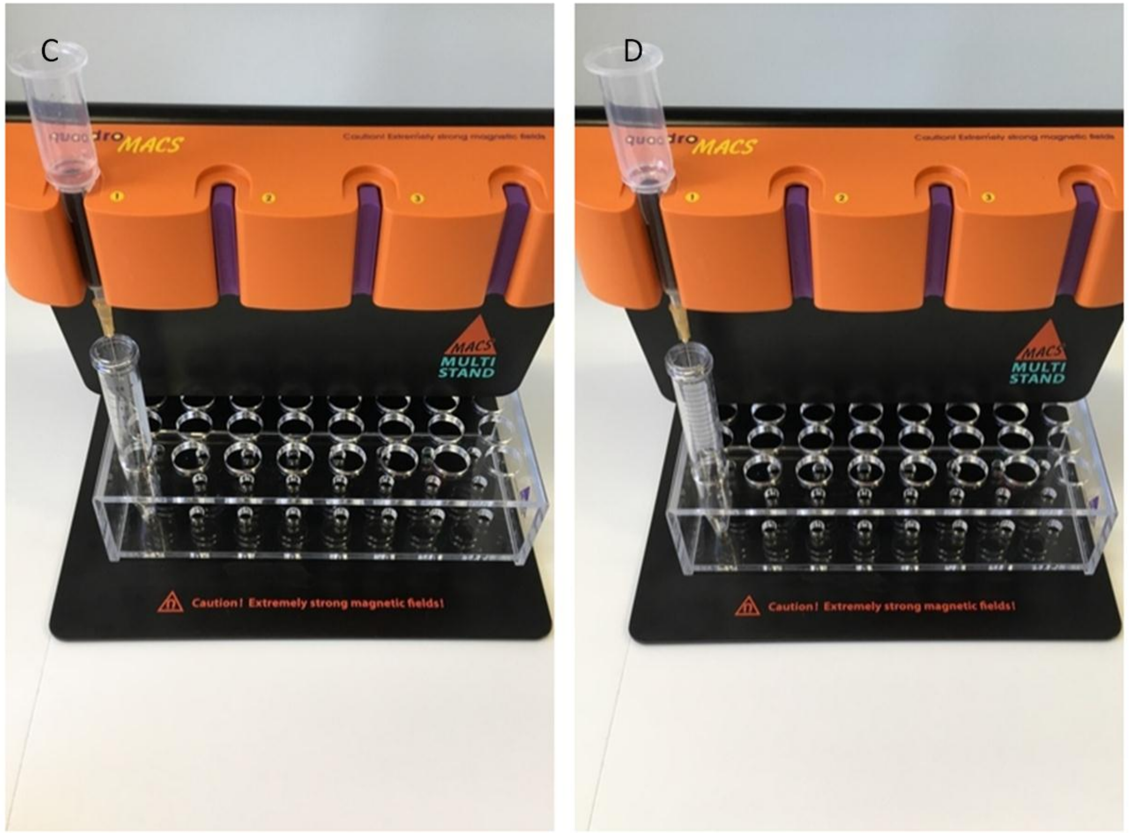
C
quadroMACS
Caution! Extremely strong magnetic fields!
MACS
MULTI
STAND
Caution! Extremely strong magnetic fields!
D
quadroMACS
Caution! Extremely strong magnetic fields!
MACS
MULTI
STAND
Caution! Extremely strong magnetic fields!

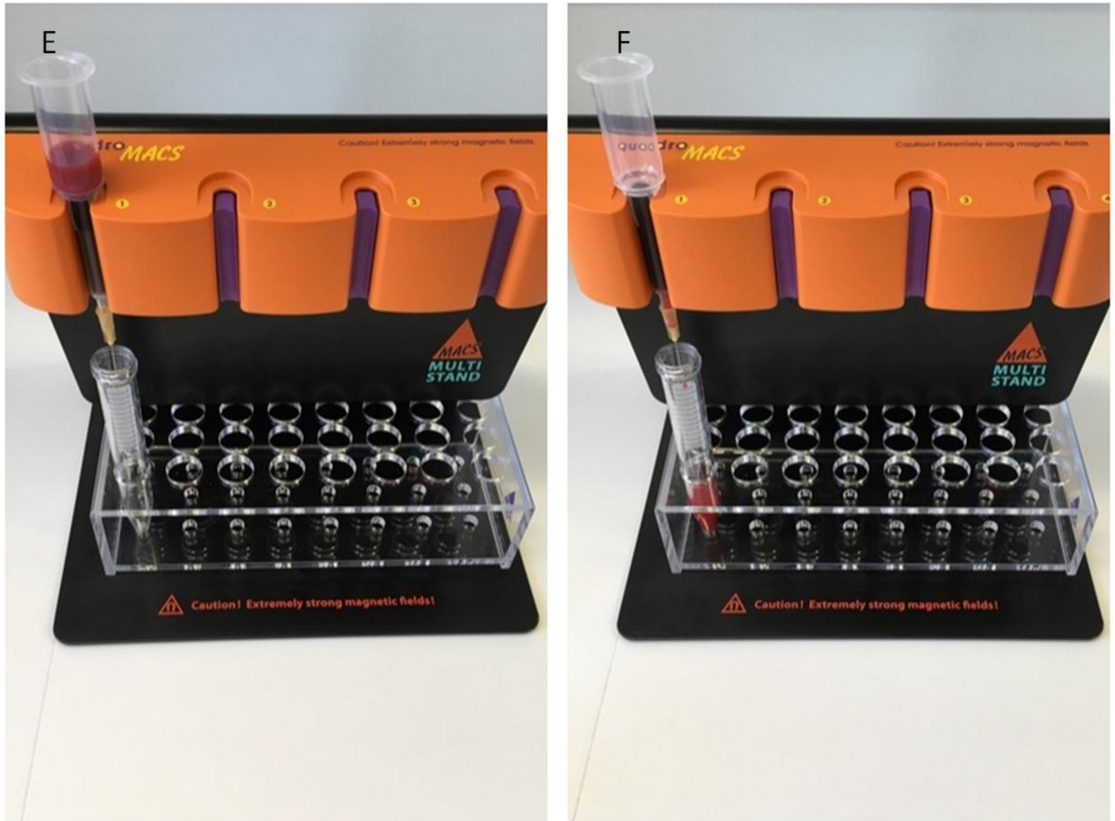
E
F
quadroMACS
MACS
MULTI STAND
Caution! Extremely strong magnetic fields!

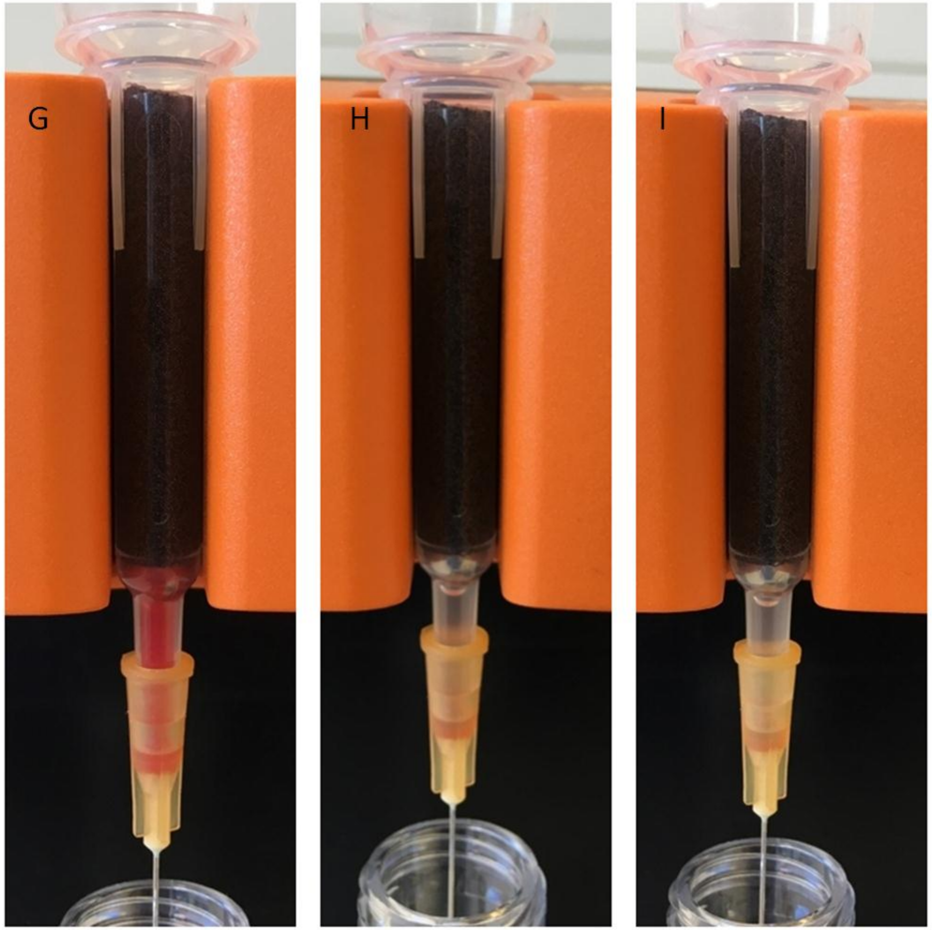
G
H
I

Supplemental Figure S3. qRT-PCR standard curves

Standard qRT-PCR curves for detection of male gametocytes (PfMGET, texas red) and female gametocytes (CCp4, FAM) obtained using 10 fold dilutions of cultured gametocytes.

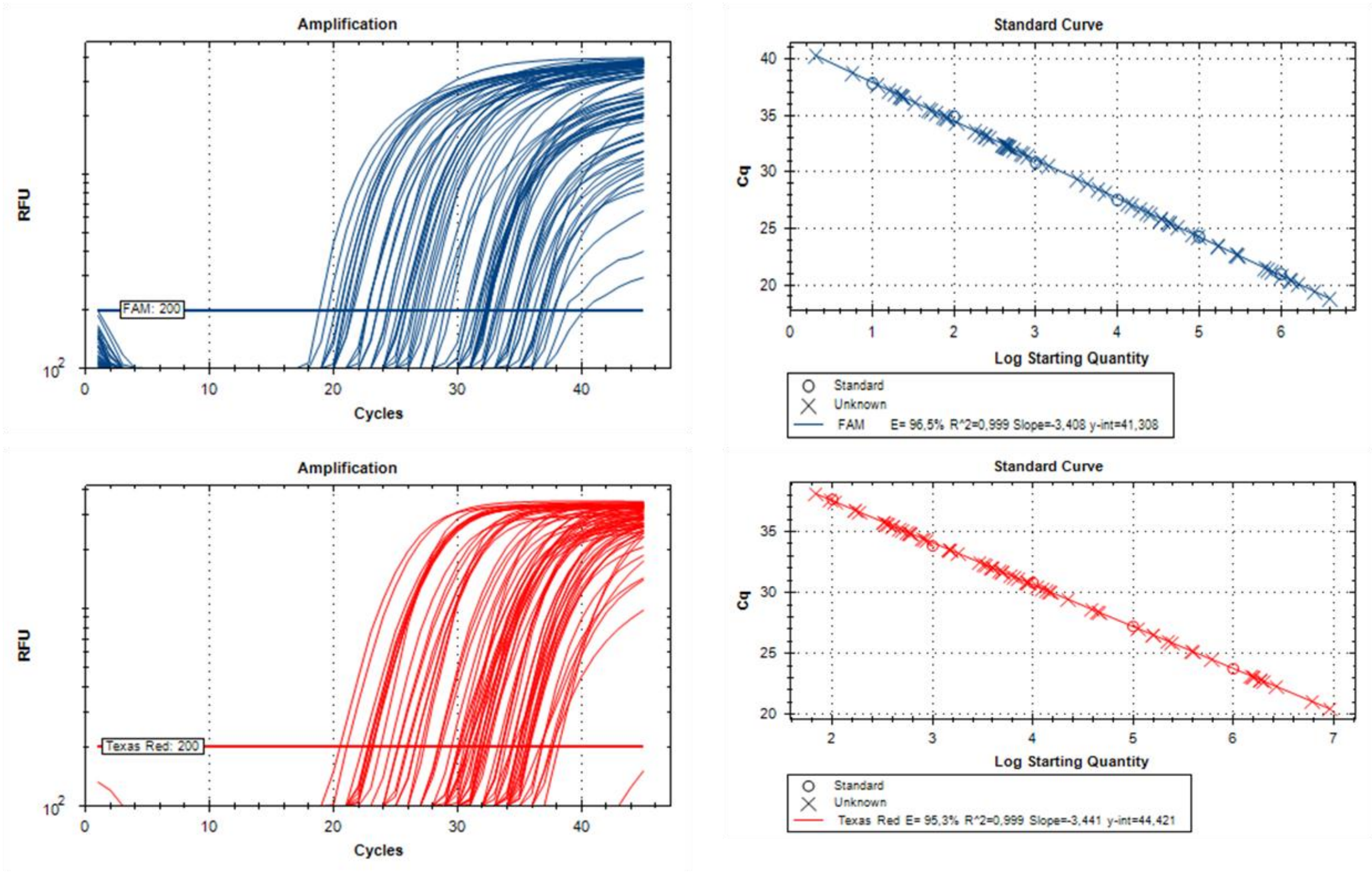

Supplement: Supplementary file 1 [file tpmd180773.SD1.pdf]
